# Environmental Impact Assessment of the Industrial Estate Development Plan with the Geographical Information System and Matrix Methods

**DOI:** 10.1155/2012/407162

**Published:** 2012-01-05

**Authors:** Mohammad Ghasemian, Parinaz Poursafa, Mohammad Mehdi Amin, Mohammad Ziarati, Hamid Ghoddousi, Seyyed Alireza Momeni, Amir Hossein Rezaei

**Affiliations:** ^1^Tehran Wastewater Company, Power Ministry, Tehran, Iran; ^2^Environment Research Center, Isfahan University of Medical Sciences, Isfahan, Iran; ^3^Department of Geography, Isfahan University, Isfahan, Iran; ^4^Environment Division, Isfahan Industrial Estates Company, Isfahan, Iran; ^5^Science and Research Branch, Islamic Azad University, Tehran, Iran

## Abstract

*Background*. The purpose of this study is environmental impact assessment of the industrial estate development planning. *Methods*. This cross-sectional study was conducted in 2010 in Isfahan province, Iran. GIS and matrix methods were applied. Data analysis was done to identify the current situation of the region, zoning vulnerable areas, and scoping the region. Quantitative evaluation was done by using matrix of Wooten and Rau. *Results*. The net score for impact of industrial units operation on air quality of the project area was (−3). According to the transition of industrial estate pollutants, residential places located in the radius of 2500 meters of the city were expected to be affected more. The net score for impact of construction of industrial units on plant species of the project area was (−2). Environmental protected areas were not affected by the air and soil pollutants because of their distance from industrial estate. *Conclusion*. Positive effects of project activities outweigh the drawbacks and the sum scores allocated to the project activities on environmental factor was (+37). Totally it does not have detrimental effects on the environment and residential neighborhood. EIA should be considered as an anticipatory, participatory environmental management tool before determining a plan application.

## 1. Introduction

The existing tendency of industrialization and urbanization in developing countries has an enormous impact on natural and man-made environments. Pollution sources increase with the development of cities and cause contamination of air, water, and soil. Lack of urban environmental planning and management strategies has led to better concern for upcoming urban expansion [1].

Unprecedented growing rates of global human population and urban development make tremendous stress on local, regional, and global air and water quality. A necessity to better understanding of the factors that mediate the interactions between urbanization and variations of environmental quality exists [2].

Land use modification, urbanization, and infrastructure developments specifically could destruct the natural environments and are threating the biodiversity. Tools and measures must be adapted to evaluate and remedy the potential effects on biodiversity caused by human activities and developments. Within physical planning, environmental impact assessment (EIA) plays important roles in the prediction and assessment of biodiversity-related impacts from planned developments [3].

EIA is one of the main legislative tools recognized to reduce an anthropogenic impact on the environment. EIA can be defined as “a process by which information about the environmental effects of a project is collected, both by the developer and from other sources, and taken into account by the relevant decision-making body before a decision is given on whether the development should go ahead.” [4].

The purpose of EIA is to ensure that the environmental effects of a proposed development are fully considered, together with its economic or social benefits. This should be considered before the planning application would be determined. EIA is thus an anticipatory, participatory environmental management tool.

The extensive understanding of EIA as an anticipatory environmental management tool has made a significant consideration over the extent to which it is achieving its purposes. This has been measured in terms of EIA “effectiveness,” especially as discussion has moved away from issues of procedural operation to the more practical goals of EIA and its place in more comprehensive decision-making situations [5].

Geographical information systems (GISs) bring the opportunity to enhance predictable evaluation techniques (e.g., matrix-based assessments). It acts as graphic mediators of spatial knowledge and by providing an effective tool for the spatial and temporal analysis of environmental impacts. GIS has the potential to increase the objectivity and accuracy of the assessment, to improve both the understanding of environmental and planning concerns and the distribution of information. Therefore, it may help to develop the effectiveness of strategic environmental assessment practice [6].

For monitoring industrial pollution in the case of developing countries, the design of policy instruments is a demanding task. In principle, the regulator has a collection of physical, legal, financial, and other tools. However, existence of a great number of small-scale industries and informal region pollution sources, requiring knowledge, funds, technology, and skills to treat their effluent, leads to failure [7].

At the present time, due to the inappropriate expansion of industries in Iran, similar to other developing countries, environmental attitudes for suitable protection of environment are vital for next generations, and it has attracted authorities' attention. In this regard, the government has attempted to establish and to develop different industrial estates in various parts of the country for managing industrial activities to control the environmental pollution [8].

The environmental impacts of projects or actions generally include a comprehensive range of impacts. All these impacts vary in magnitude, as well as in their beneficial or adverse organization [9].

According to the industrial estates company's policy in Iran, planning the industrial units follow specific patterns and in each estate separated sites have been predicted for different industries. Therefore, each industry could be a potential source of solid, liquid, and gaseous emissions and their effects on humans, natural flora, air, soil, water sources, climate conditions, cultural heritage, and valuable materials should be evaluated.

The purpose of this study is to establish EIA for an industrial estate development planning in Iran by using the GIS and matrix methods.

## 2. Materials and Methods

This cross-sectional study was conducted in 2010, in Isfahan province, in central Iran. The following methods were used for EIA.

### 2.1. GIS Assessment Method

Environmental evaluation of Koohpayeh industrial estate by using GIS was conducted by the following processes.

#### 2.1.1. Identifying Effective Factors in Environmental Degradation

Including climate, geology, hydrology data, and some degradation factors in the region such as its location, different types of pollutants, land use, and ecological data.

#### 2.1.2. Collecting and Entering Data

The collection of information on the site and surroundings of the proposed development (“baseline” information) is essential in EIA, as in the implementation of any proposed development.

#### 2.1.3. Data Analysis

Required data for analyzing maps of different organizations was gathered with scale of 1 : 50000 and using the Universal Transverse Mercator (UTM) system, and they were given digits with ARC GIS software. In analyzing steps, data analysis was done by using existing operators to identify the current situation of the region, zoning vulnerable areas, and scoping the region, which is affected by pollutants. For this purpose, overlay method and analysis of ground water was used.

To perform zoning, considered parameters were selected and then they were scored by expert evaluators. Thereafter, classified layer zones were categorized.

### 2.2. Evaluation with Quantitative Method by Using the Matrix

Quantitative evaluation method was done by using the matrix of Rau and Wooten [9], that is, the other format of Leopold Matrix (1).

(1) $$\begin{array}{cc} \text{Net}\;\text{score}\;\text{for}\;\text{impact} & =\text{magnitude}\;\text{of}\;\text{effect}\\ & \;\times \left(\text{importance}\;\text{of}\;\text{effect}\right)\text{.} \end{array}$$

In each project, the effect magnitude of activities is defined based on environmental parameters with classifying each group of pollutants; for example, it is defined based on technical and scientific principles for determining effect magnitude of each group. The scope for importance of effect is similar for all of the impacts. In this study, the range of importance of effect is defined with the numbers of 0 to 5 as it presented in Table 4. 

### 2.3. District of the Study Area

**Figure 1:**
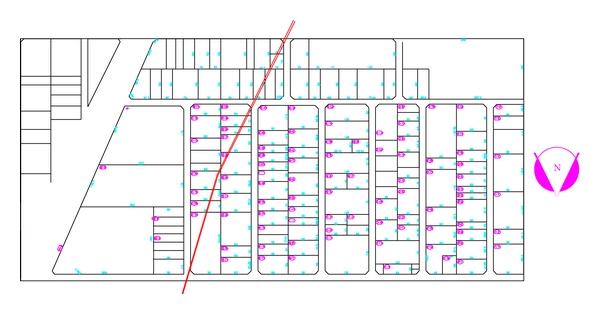
Current and development layout of industrial zones in the study project.

In this study, development planning of the industrial estate, located in the Isfahan province, central of Iran, was evaluated. The current area of the industrial estate is 150 hectares (Figure 1, dark color part), and its extent development planning is 350 hectares. The development plan is included various types of industries such as food, chemical, ceramic industries, thermal and sound insulation, and other manufacturing industries (Figure 1). 

## 3. Results

The entries in matrix represent not only an indication of the areas impacted by each action, but also serve as a measure of the impact's extent.  Table 1 provides an illustration of the basic structure of the matrix method approach, namely, a matrix in which each proposed action (or its separate components) is identified as a column of the matrix and the environmental conditions or impacted areas are identified as the rows of the matrix.

### 3.1. EIA for Gaseous and Particulate Pollutants via Matrix

#### 3.1.1. Qualitative Analysis

**Figure 2:**
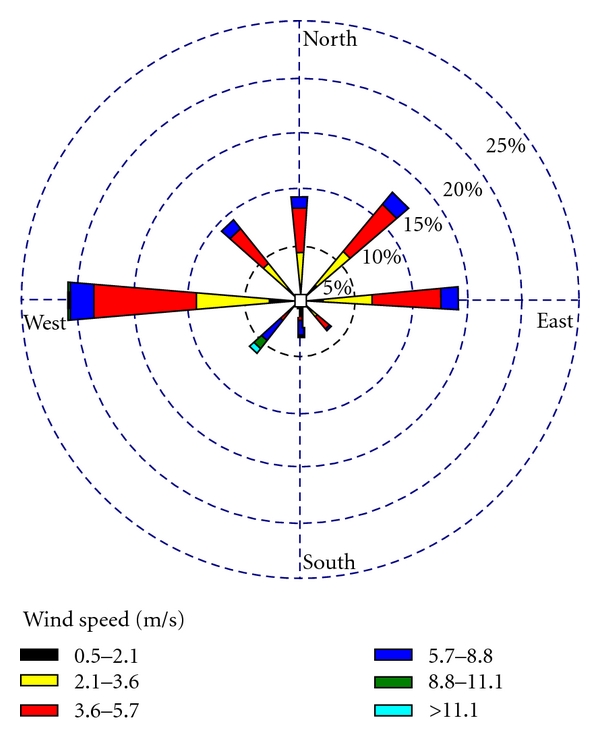
Wind rose of the study area.

Based on daily meteorological data in synoptic station of Naein city in a one year period (2010), the minimum and maximum speed of prevailing wind were 0 and 15 m/s, respectively. Moreover, most days in a year have a mild air flow. So, Koohpayeh region is in class C of atmospheric stability classes and the region atmosphere is slightly unstable. Therefore, the particulate and gaseous pollutants will become diluted and their negative effects would reduce. However, the wind rose of the region (Figure 2) shows that the prevailing wind in this industrial estate has east-west direction and vice versa with 21% of region wind rose. There is no residential area in downstream, and air pollutants have enough opportunity for becoming diluted in the environment. Instability air in industrial estate location cause releasing the emissions and it increases the concentration of air pollutants in comparison with background air.

#### 3.1.2. Quantitative Analysis

Given the mentioned conditions, the importance of particulate and gaseous pollutants on this industrial estate air quality is very low with the score of 1 (Table 1). Moreover, considering the atmospheric stability, the magnitude of effect for C stability class is −3 (Table 5). Therefore, as shown in Table 1, cell (4, 11), the net score for impact of “industrial units operation” on “air quality” of the project area is equal to −3  [1 × (−3) = −3].

The magnitude effect of particulate and gaseous pollutants on air quality based on atmospheric stability classes is determined in Table 5.

### 3.2. Air Pollution Assessment through GIS

Considering the highest percentage wind speed of 2–5 m/s in the study area with an average speed of 3 m/s, the height of 25 m for the most elevated stack in the estate, and using Gaussian model, pollutants concentration in various distances from the estate is indicated in Table 2.

**Figure 3:**
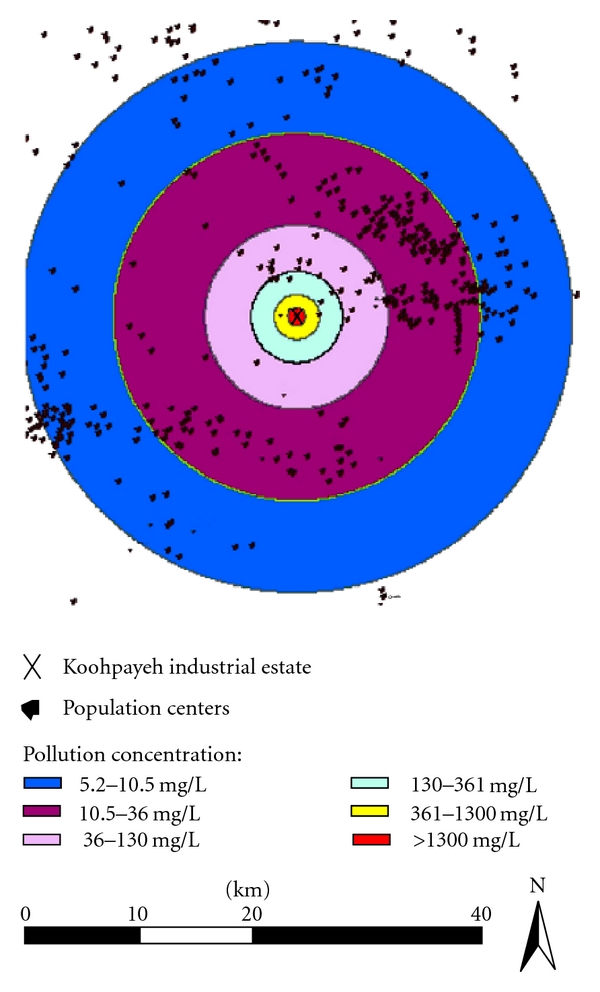
Pollutant emission on the residential districts of the study area.

According to the transition of industrial estate pollutants, residential places located in the radius of 2500 meters of the city would be affected more (Figure 3).

### 3.3. Gaseous and Particulate Pollutants Effects on Plant Species

The status of pastures has been selected as a criterion of evaluating pollutants effects on plants. Due to severe destruction of soil and vegetation in the pastures, five classes were considered for rating. For this purpose, rangeland vegetation percentage method is provided by US Range service.

In this method, current rangeland composition vegetation of increaser and decreaser plants are calculated in climax stage, and their statuses will be determined by using 5 class- scales in Table 6.

#### 3.3.1. Quantitative Analysis

Excavation and embankment, construction of industrial units, water distribution network, and industrial wastewater collection system have a low magnitude effect with score of −2 (Table 7) and very low importance impact with score of 1 (Table 4) was considered. Therefore, as shown in Table 1, cell (6, 6), the net score for impact of “Construction of industrial units” on “Plant species” of the project area is equal to −2  [1 × (−2) = −2].

### 3.4. EIA of the Wildlife Zones

**Figure 4:**
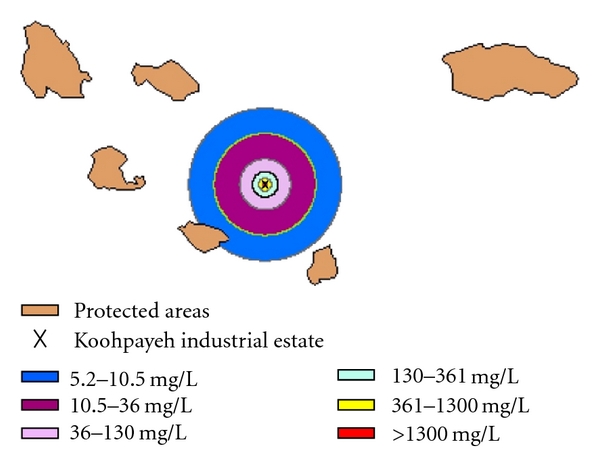
The magnitude of air pollution effects on wildlife protected zones of the study area.

Environmental protected areas are not affected by the air and soil pollutants because of their distance from industrial estate. According to the location of the wildlife protected areas (southwest of the estate) and region wind rose (Figure 2), the percentage of wind to the southwest about 6 percent of the winds, blow into this region during the year. Therefore, these regions were not highly affected by pollutants. The pattern of prevailing winds in this estate indicated that these winds have west-east direction.  Figure 4 shows the magnitude of air pollution effects on wildlife-protected areas.

### 3.5. EIA for Groundwater Quality via Matrix

#### 3.5.1. Quantitative Analysis

As indicated in Table 1, cell (2, 10), importance effect of discharge of industrial effluents on ground water considered “low” with the score of 2, and medium and intermittent magnitude of effect considered with the score of −3 (Table 3).

Water resources of study area are provided by wells and Qanats, and there is no surface water resource in this area. Therefore, for recognizing water resources exposed to pollution sources of estate only position and direction of groundwater flow was determined. Groundwater resources are located in the west, south, southwest, northeast, and northwest of the industrial estate.

**Figure 5:**
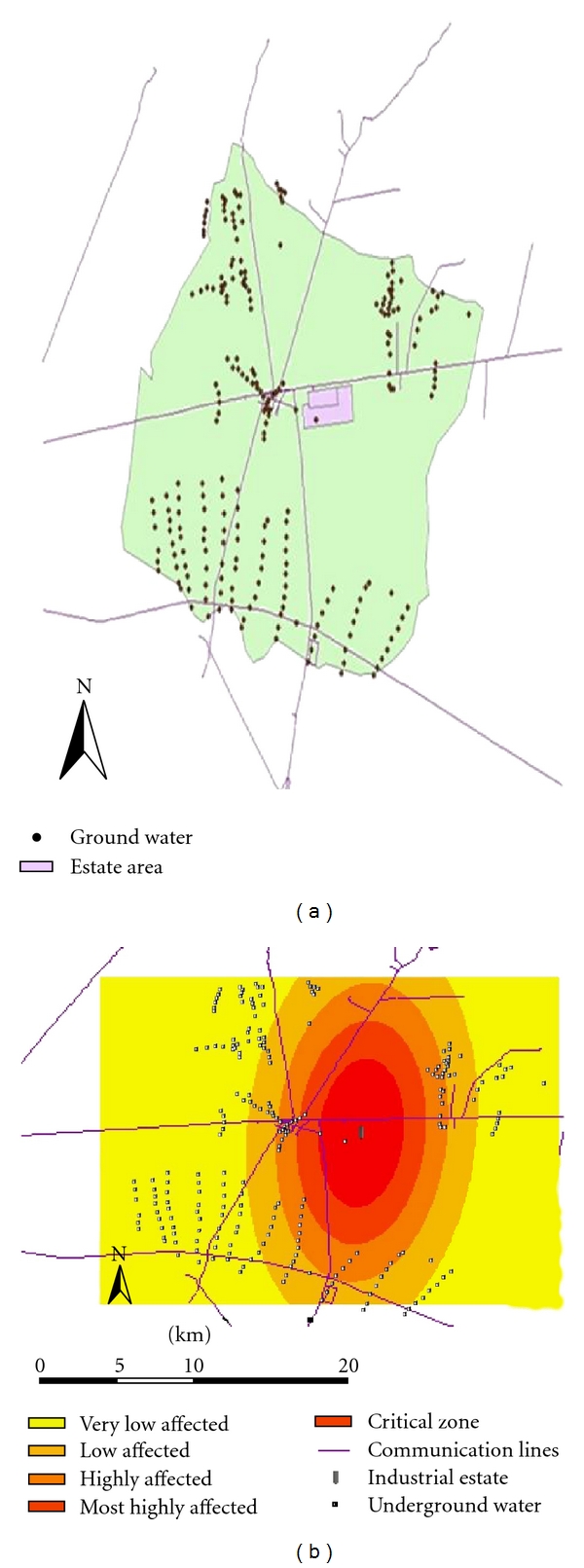
The position of the groundwater resources to the industrial estate (a) and the Qanats exposed to pollution (b).

The flow direction of all groundwater resources was south and southwest. The position of groundwater resources to the industrial estate was depicted in Figure 5.

## 4. Discussion

This study found that more attention should be paid to the regions located in the zones with very high, high, and medium vulnerability than to other regions. These regions consisted of Koohpayeh city; the Qanats located in south and south west of the industrial estate; gardens located in suburbs; the residential places that are located in the east of the industrial estate.

According to the transition of industrial estate pollutants, residential places located in the radius of 2500 meters of the city will be affected more. Koohpayeh city, which is the most populated center in this region, is more vulnerable because of its location, thus it needs more attention than other regions.

Regarding the natural environment aspects of conducting the project, the largest percentage of land use is related to the low-density pastures and the lowest percentage is devoted to residential areas.

The impact assessment is a management tool for stakeholders and decision makers; it serves as a supplementary tool for other engineering studies and economic projects.

Industrial ecosystem is an important approach for sustainable development. In an industrial environment, a group of industries are interconnected through mass and energy exchanges for mutual benefits. However, some mass and energy exchange activities may have unexpected environmental impact [10].

Industrial development could be defined as providing the foundation for industrial expansion and social stability with reducing the environmental destructive impacts. The necessity to achieve mentioned goal is to merge environmental concerns with different levels of policy making and controlling levels [8].

To predict, identify, and determine accurate analysis of positive and negative effects of an environmental project on natural and man-made environments, it is necessary to evaluate these projects before their implementation to estimate the minimum negative consequences in the future. Thus, spatial analyzing tool can be used under water analyzing tool and destruction model in GIS.

The purpose of using these tools is preventing degradation and reduction of vulnerability level of ecosystems as well as prevention of the destruction in development programs. Moreover, some preventive ways against the short-term recurrence of destruction can be suggested. Therefore, it is vital to evaluate the environmental impacts of the industrial development to provide a clear management for the decision-makers and stakeholders. Environmental protection issues are considered as parts of the national laws, and application of such projects may be of help in this regard.

The main limitation of this study is its cross-sectional nature. Some damages caused by this development project such as groundwater contamination may have nonmeasurable environmental impact, which is not compensable.

## 5. Conclusion

Results of quantitative analysis of the effects of environmental factors on the industrial estate development project by the matrix method demonstrated that the sum scores allocated to the project activities on environmental factor is “+37,” which means that positive effects of project activities outweigh the drawbacks and totally it does not have detrimental effects on the environment and residential neighborhood (Table 8).

Given that the qualitative and quantitative analysis, industrial estate development project might have some negative effects on some environmental factors but generally, development of this estate should not be prevented. Moreover, with considering all factors including socio-economic factors that have special effect on development process, performing of the project with minimum negative consequences should be provided.

## Figures and Tables

**Table 1:** Matrix for EIA of the industrial estate.

	Environmental factors											
Project Activities		Soil and Earth	Ground water quantity and quality	Flood	Air quality	Noise	Plant and animal species	Occupation	Housing, education, and welfare	Emigration	Health and safety	Total Factor Impact
		1	2	3	4	5	6	7	8	9	10	**11**

Excavation and embankment	1	−2(2)	0	1(2)	−1(2)	−1(1)	−2(1)	1(3)	0	0	0	**−2**
Construction of water distribution network	2	−1(1)	−2(1)	0	−1(2)	−1(1)	−2(1)	1(3)	1(2)	0	−1(1)	**−4**
Construction of storm water and industrial sewer	3	−1(1)	−1(1)	2(3)	−1(2)	−1(1)	−2(1)	1(3)	1(2)	0	−1(1)	**3**
Construction of industrial wastewater treatment plant	4	−1(3)	0	0	−1(2)	−1(1)	−2(1)	1(3)	1(2)	0	−1(1)	**−4**
Transportation of workers	5	0	0	0	0	0	0	1(5)	0	0	0	**5**
Construction of industrial units	6	−1(1)	0	0	0	−1(1)	−2(1)	1(3)	1(2)	0	−1(1)	**0**
Industrial Solid waste disposal	7	−2(2)	−3(2)	0	0	0	0	0	0	0	−1(1)	**−11**
Required water (Industrial use)	8	0	−2(3)	0	0	0	0	0	0	0	0	**−6**
Operation of wastewater treatment plant	9	1(1)	−1(2)	0	−2(1)	0	0	0	−1(1)	0	2(3)	**0**
Discharge of Industrial effluent	10	−2(1)	−3(2)	0	0	0	2(4)	0	0	0	0	**0**
Industrial units operation	11	−2(3)	0	0	−3(1)	−2(1)	−1(1)	5(5)	5(5)	4(5)	−2(1)	**56**

Total action impact	**12**	**−21**	**−23**	**8**	**−13**	**−7**	**−3**	**45**	**32**	**20**	**−1**	**37**

A negative sign (−) in the front of the magnitude number shows that the impact is adverse (Table 2).

**Table 2:** Predicted pollutants concentration in various distances from the estate using Gaussian model.

Distance (km)	Pollutants concentration (mg/L)
2	1300
5	361
10	130
20	36

**Table 3:** Groundwater pollution potential by the industrial wastes.

	Pollution level		
	Low	Medium	High
Continuous	Leakage of sewers and industrial wastewater treatment units	Leachate percolated from Industrial wastes landfills	Leakage of Industrial reactors, underground and aboveground reservoirs
Intermittent	Leakage of Industrial sites	Discharge of Industrial effluents	
Accidental			Suddenly and severe spills

**Table 4:** Scope for importance of the effect for all of the impacts of the project.

Importance of effect	Score
No effect	0
Very low effect	1
Low effect	2
Important effect	3
Very important effect	4
Extremely important effect	5

**Table 5:** Ranking of magnitude effect on atmospheric stability classes.

Effect magnitude	Score
Class A: extremely unstable	1
Class B: unstable	2
Class C: slightly unstable	3
Class D: neutral	4
Class E: slightly stable	5
Class F: stable to extremely stable	6

**Table 6:** Classification of pastures' status.

Status	Vegetation composition percentage in climax stage
Excellent (E)	81–100
Good (G)	61–80
Fair (F)	41–60
Poor (P)	21–40
Very poor (VP)	<20

**Table 7:** Magnitude effect of the industrial estate on plant species according to pastures' status.

Status	Magnitude of effect
Very low (very poor)	1
Low (poor)	2
Moderate	3
High (good and excellent)	4

**Table 8:** Effect magnitude of project activities on groundwater quality.

Effect magnitude	Score
No effect	0
Low and intermittent	1
Low and continuous	2
Medium and intermittent	3
Medium and continuous	4
High and continuous	5
